# Implementing video-based group music therapy during cancer treatment: insights from a mixed-methods study

**DOI:** 10.1007/s00520-026-10601-5

**Published:** 2026-03-25

**Authors:** Miriam Grapp, Charlotte Flock, Hans-Christoph Friederich, Till Johannes Bugaj

**Affiliations:** 1 Department of General Internal and Psychosomatic Medicine, University Hospital Heidelberg, Heidelberg University, Im Neuenheimer Feld 460, Heidelberg, 69120 Germany; 2 National Center for Tumour Diseases (NCT), Heidelberg University Hospital, Heidelberg University, Heidelberg, Germany; 3 DZPG (German Centre for Mental Health – Partner Site Heidelberg/Mannheim/Ulm), Heidelberg, Mannheim, Ulm, Germany

**Keywords:** Music therapy, Video-based interventions, Supportive care, Cancer treatment, Mixed-methods study

## Abstract

**Purpose:**

Music therapy is a beneficial supportive intervention in oncology. However, many patients undergoing cancer treatment face treatment-related physical impairments that limit participation in in-person sessions. This study explores the implementation of a video-based group music therapy intervention for patients undergoing tumour therapy.

**Methods:**

A mixed-methods, single-arm study was conducted with 40 adult cancer patients. The intervention consisted of eight 90-min video-based group music therapy sessions. Standardized questionnaires and open-ended questions were used pre- and post-intervention. Therapists provided structured feedback after each session. Evaluation was guided by the RE-AIM framework (Reach, Effectiveness, Adoption, Implementation, Maintenance).

**Results:**

Recruitment was efficient, with 40 of 44 screened patients enrolled. Attendance (90.1%) and completion (87.5%) rates were high. Participants rated the intervention as highly helpful (*M* = 4.77, SD = 0.49), with significant reductions in depression and anxiety (*p* < 0.001). In their feedback, patients frequently reported shared musical experiences and a sense of emotional closeness as particularly meaningful aspects of the sessions. Therapists observed strong group cohesion despite initial technical challenges. Follow-up data indicated continued use of music in daily life, suggesting lasting impact.

**Conclusion:**

Video-based group music therapy is a feasible and well-accepted intervention for patients undergoing cancer treatment. It addresses access barriers due to treatment-related physical impairments and supports continuity of care. The intervention showed promising results across RE-AIM dimensions, including symptom relief and integration into everyday life. Further studies should assess efficacy and explore adaptations for specific patient subgroups.

**Trial registration:**

German Clinical Trials Register (DRKS00032339) (registered August 3, 2023).

**Supplementary Information:**

The online version contains supplementary material available at 10.1007/s00520-026-10601-5.

## Introduction

Music therapy is increasingly recognized in supportive oncology, with empirical evidence supporting reductions in anxiety, depression, and pain, mitigation of treatment-related effects, and improvements in quality of life [1–3]. Recent literature conceptualizes music therapy as a low-risk, non-pharmacological adjunct within multimodal cancer care, emphasizing its role in emotional regulation, psychological coping, and patients’ subjective treatment experience across the cancer trajectory [4]. Music therapy encompasses both active and receptive formats [1]. Active approaches engage patients in the creation of music—via singing, instrumental performance, or improvisation—whereas receptive methods emphasize guided listening to live or recorded music. Music therapy can be applied in individual and group settings, depending on the treatment goal and approach. Group interventions have become an integral part of psychosocial cancer care and are an effective way of caring for a greater number of people [5]. Despite these well-documented benefits, many patients undergoing tumour treatment are unable to participate in face-to-face (group) interventions due to their compromised physical condition [6].

This gap may be addressed through eHealth interventions or video-based counseling and therapy, which have gained legitimacy and prominence during the COVID-19 pandemic [7]. These modalities significantly enhance accessibility, particularly for individuals residing in geographically remote areas or experiencing physical impairments [8]. However, challenges such as limited digital literacy and the need for patients to manage their therapeutic setting independently must be addressed [9]. Online group interventions present specific challenges, as the therapist’s physical presence—crucial in face-to-face settings for shaping group dynamics, providing structure, and offering nonverbal regulation—is absent and must be replaced by alternative forms of guidance and containment [10].

The COVID-19 pandemic has expedited efforts to transfer existing music therapy concepts to the online setting [11]. A growing body of literature supports the feasibility of online music therapy—both individual and group-based—across various clinical contexts and populations, including neurological disorders, palliative care, chronic pain, spinal cord injuries, dementia, and psychologically distressed university students [12–19]. However, despite the extensive research on face-to-face music therapy in oncology, the literature on online music therapy in this field remains sparse [20]: Rabinowitch et al. [21] conducted a mixed-methods study evaluating a one-hour online group music listening session for patients undergoing cancer treatment. Folsom et al. [22] and Avery et al. [23] investigated the adaptation of a face-to-face group music therapy to an online format through a case study and a qualitative study respectively, one within an integrative oncology setting and the other for young adults (AYAs) with cancer.

Against this background, we developed a video-based group music therapy intervention specifically tailored to the needs of patients undergoing tumour therapy. Rather than adapting an existing face-to-face format, this intervention was developed specifically for the online setting. The aim of the present single-arm mixed methods study was to examine the early-stage implementation of this novel intervention in the context of routine psychosocial support during cancer treatment. We examined how the intervention could be implemented within the context of tumor therapy, which factors influenced its practical use, how it was received and valued by patients, and to what extent it could be integrated into both treatment routines and everyday care.

## Methods

The evaluation was primarily structured using the Reach, Effectiveness, Adoption, Implementation, and Maintenance (RE-AIM) framework [24]. Reporting followed the Good Reporting of A Mixed Methods Study (GRAMMS) [25] criteria and the Reporting Guidelines for Music-Based Interventions (RG-MBI) [26]. Summary tables of the GRAMMS and RG-MBI guidelines are available as supplemental material (Online Resources 1 and 2).

### Study design

A single-center, single-arm, concurrent mixed-methods study was conducted in a real-world setting, applying an exploratory design. Quantitative and qualitative approaches were integrated to yield a comprehensive understanding of the implementation process of the video-based group music therapy intervention. The term “concurrent” refers to the simultaneous collection and analysis of quantitative and qualitative data, which were processed independently and integrated during the interpretation of results. The study adhered to the Declaration of Helsinki and received ethical approval from the Ethics Committee of the University of Heidelberg (S-767/2022). It was registered in the German Clinical Trials Register (DRKS00032339).

### Participants, recruitment, and procedure

Eligible participants were adults undergoing tumor therapy (chemotherapy, radiotherapy, or immunotherapy), irrespective of tumor type, disease duration, or treatment intent (curative or palliative). Exclusion criteria included severe psychiatric conditions (e.g., suicidality or dissociative disorders), acute psychological crises, or inadequate internet access. Recruitment took place via flyers and social media. Interested individuals contacted the study team and took part in a structured online pre-interview to assess their suitability for participation. Participants provided written informed consent. Questionnaires were completed before the intervention (T0), immediately after (T1), and 12 weeks later (T2).

### Intervention

**Fig. 1:**
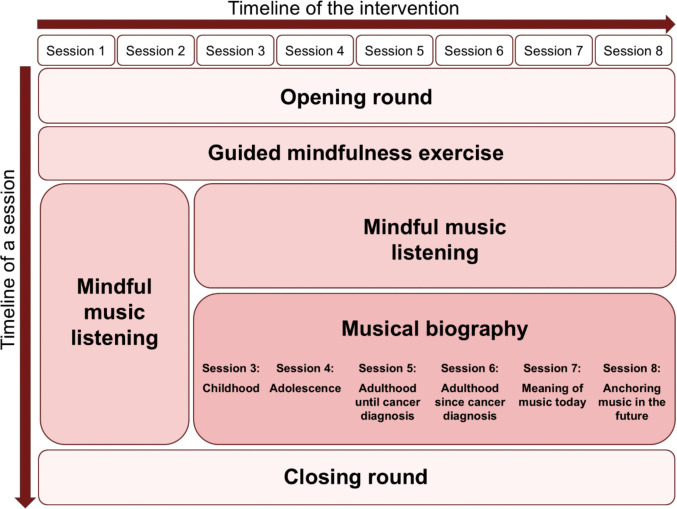
Structure and elements of the video-based group music therapy intervention

The video-based group music therapy draws on two main approaches: (1) Regulative Music Therapy (RMT), which fosters an accepting, benevolent attentiveness to both the music and the inner processes it evokes [27], and (2) a biographical approach that uses individuals’ lifelong musical experiences as personal coping resources [28, 29]. It comprises eight weekly 90-min sessions conducted via a secure, institutionally hosted video conferencing platform (recommended group size: eight participants). Sessions are co-facilitated by a music therapist (also trained as a music educator and certified psycho-oncologist) and a psychotherapist with psycho-oncological experience. Technical support is provided by a psychology student. Prior to the group sessions, each participant attends an individual video session to familiarize themselves with the platform, clarify the structure of the intervention, and discuss requirements for the online setting—especially the need for a quiet, undisturbed environment. Intervention fidelity was ensured through manualized protocols and structured monitoring. The intervention comprises the following components (see Fig. 1):

- ***Opening and closing round:*** At the beginning and end of each session, participants share their current emotional state, mood, and thoughts.
- ***Guided mindfulness exercise:*** A guided mindfulness exercise, adapted to the online setting, was used to help participants settle into the virtual environment and prepare for the subsequent intervention elements. The exercise focused on present-moment awareness and was conducted without music.
- ***Mindful music listening:*** Patients are guided to listen to the music mindfully, paying attention to their physical, emotional, and cognitive responses. Initially, pieces from the classical music repertoire selected by the therapists are used. In later sessions, participants introduce personally meaningful pieces, with no restrictions on musical style (see Online Resource 3 for an overview of music pieces used during therapy sessions). Music is shared via a platform link (e.g., cloud storage). At a coordinated signal, participants listen simultaneously yet individually on their own devices. Each listening phase is followed by group discussion and reflection.
- ***Musical biography*:** The musical biography is a biographical method that explores music’s personal significance in patients’ lives – especially during challenging times – and supports them in recalling and reconnecting with meaningful pieces. Each session focuses on a specific life stage and the role of music during that period. Using reflective questions and worksheets, participants reflect on their musical past, first in small breakout groups, then with the full group.

### Outcomes

Outcomes were assessed along the five RE-AIM dimensions:

- ***Reach*** was evaluated based on recruitment efficiency (time to target sample; numbers invited and enrolled), participation (enrollment and attendance), and participant characteristics, including sociodemographic and clinical variables. Barriers to participation (e.g., limited internet access and treatment-related burden) were documented during recruitment.
- ***Effectiveness*** was assessed using a multi-method approach: a post-intervention Likert scale on perceived helpfulness (1 = “not at all helpful” to 5 = “extremely helpful”), an open-ended question on the most beneficial components (T1), and standardized questionnaires at T0, T1, and T2. These included the PHQ-9 (depression) [30], GAD-7 (anxiety) [31], and MDASI (physical symptoms and daily life interference) [32].
- ***Adoption*** was not formally measured but inferred from contextual observations, such as institutional support and integration into existing psycho-oncology services.
- ***Implementation*** was evaluated via completion rate, dropout reasons, and adherence (session attendance, reasons for absence). At T1, participants answered open-ended questions on suggestions for improvement and perceived benefits and challenges of the online format (see Online Resource 4 for the full question set reflecting RE-AIM dimensions Effectiveness and Implementation). Therapists documented session impressions in structured reports, capturing positive developments, challenges, and group dynamics.
- ***Maintenance*** was assessed through a follow-up questionnaire on sustained perceived benefit (Likert scale) and open-ended feedback on long-term effects and everyday use of intervention elements. Standardized outcomes (PHQ-9, GAD-7, and MDASI) were re-assessed to evaluate the persistence of effects.

### Sample size and statistical analyses

Given the exploratory nature of the study, no formal sample size calculation was conducted. Initially, 24 patients (three group cycles) were planned. Due to unexpectedly high interest and efficient recruitment, the sample was increased to 40 patients (five cycles) prior to data analysis. Quantitative data were analyzed using IBM SPSS (Version 29). Descriptive statistics summarized adherence, completion, retention, recruitment, and rating scale outcomes. Although not primarily powered to detect significant changes, paired-samples t-tests were used to compare pre- and post-intervention scores, with Bonferroni–Holm correction and effect sizes reported as Cohen’s *dz*. Repeated-measures ANOVAs examined effect stability across T0, T1, and T2, applying Bonferroni–Holm correction across four outcomes (PHQ-9, GAD-7, MDASI severity, and MDASI interference). Statistical significance was defined a priori as *p* < 0.05 (two-tailed). Significant main effects were followed by adjusted post-hoc tests; partial eta squared (*η*^*2*^*ₚ*) indicated effect sizes. Baseline characteristics of completers and non-completers were compared descriptively due to the small number of non-completers. Dropout bias related to follow-up participation was examined separately by comparing completers with and without follow-up data on baseline characteristics using independent-samples *t*-tests for continuous variables, and either chi-squared tests or Fisher’s exact tests for categorical variables, depending on expected cell frequencies. Qualitative data from open-ended responses and therapist records were analyzed using Mayring’s qualitative content analysis [33]. Sentences served as coding units; subcategories were inductively derived and grouped into overarching themes in MAXQDA.

## Results

### Reach

**Fig. 2:**
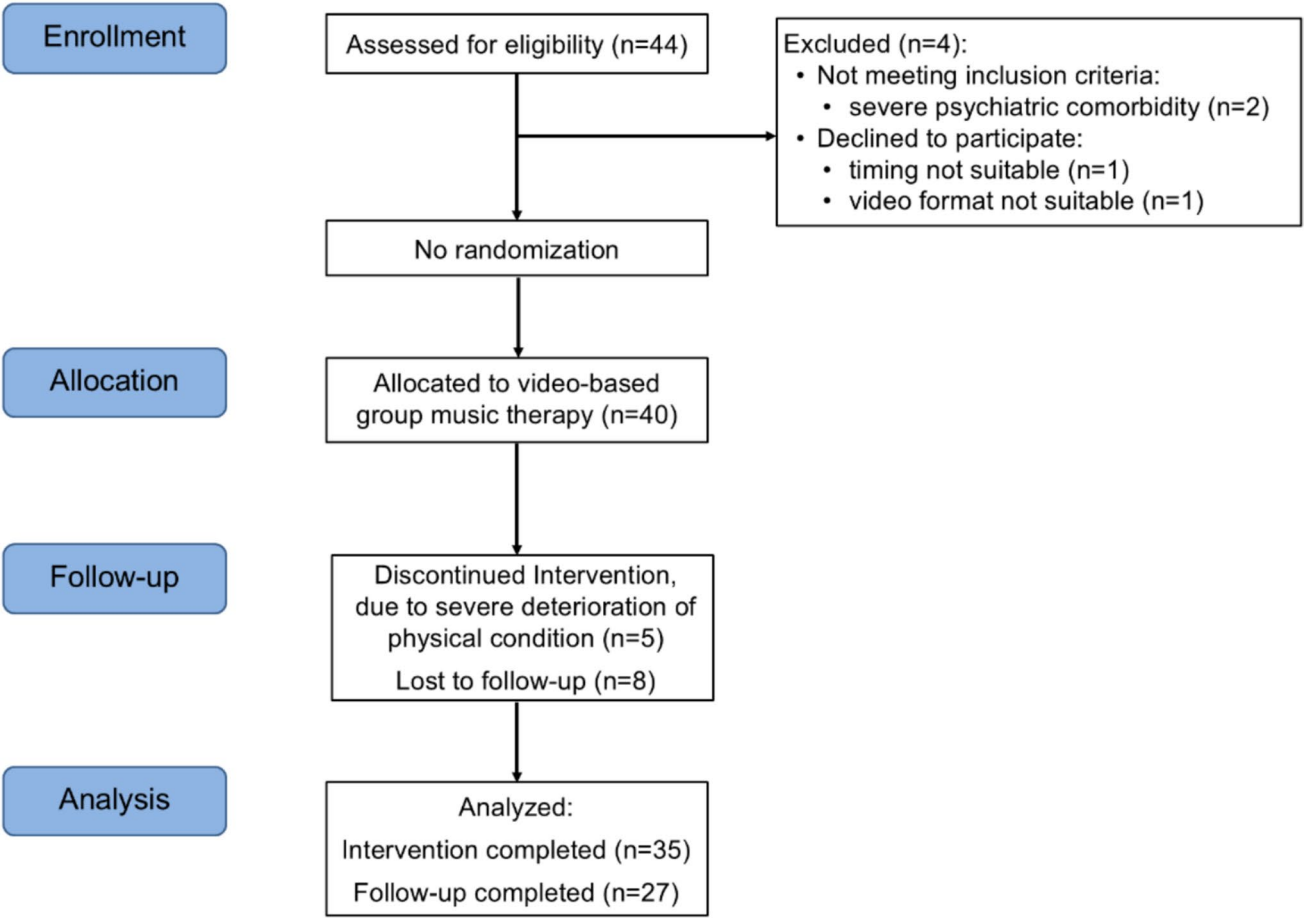
Participant flow diagram

Of 44 patients screened during preliminary interviews, 40 were enrolled in the intervention, while four were excluded (reasons detailed in Fig. 2). Recruitment took place between August 2023 and September 2024, with recruitment phases starting eight weeks before each group cycle and ending once the target size was reached. Recruitment progressed efficiently, with all planned group sizes reached within the expected timeframe. The five group cycles were conducted between October 2023 and January 2025. Demographic and clinical baseline characteristics of participants are presented in Table 1, including descriptive information for intervention completers and non-completers.

**Table 1:** Baseline demographic and clinical characteristics of the total sample, intervention completers, and non-completers

	Total sample (*n* = 40)	Intervention completers (*n* = 35)	Intervention non-completers (*n* = 5)
**Age**			
Mean (SD)	53.1 (11.7)	53.8 (12.4)	48.6 (14.2)
Range	26–81	26–81	33–66
**Gender,** *n* (%)			
Male	6 (15.0)	5 (14.3)	1 (20.0)
Female	34 (85.0)	30 (85.1)	4 (80.0)
**Relationship status,** *n* (%)			
Single	6 (15.0)	5 (14.3)	1 (20.0)
In a relationship/married	23 (57.5)	21 (60.0)	2 (40.0)
Divorced	5 (12.5)	5 (14.3)	0 (0.0)
Widowed	6 (15.0)	4 (11.4)	2 (40.0)
**Residence,** *n* (%)			
City	16 (40.0)	14 (40.0)	2 (40.0)
Rural area	24 (60.0)	21 (60.0)	3 (60.0)
**Tumor entity** (*n* (%))			
Breast	21 (52.5)	18 (51.4)	3 (60.0)
Female genitalia	5 (12.5)	4 (11.4)	1 (20.0)
Lung	4 (10.0)	3 (8.6)	1 (20.0)
Gastrointestinal tract	4 (10.0)	4 (11.4)	0 (0.0)
Skin	2 (5.0)	2 (5.7)	0 (0.0)
Male genitalia	2 (5.0)	2. (5.7)	0 (0.0)
Head and neck	1 (2.5)	1 (2.9)	0 (0.0)
**Duration of illnes**			
Mean (SD)	11.7 (8.8)	12.57 (9.1)	5.8 (3.6)
Range	2–48	2–48	2–11
**Treatment intent (***n* (%))			
Palliative	23 (57.5)	20 (57.1)	3 (60.0)
Curative	17 (42.5)	15 (42.9)	2 (40.0)
**Previous experience in group therapy** (*n* (%))			
Yes	12 (30.0)	12 (34.3)	0 (0.0)
No	28 (70.0)	23 (65.7)	5 (100.0)
**Previous experience in psychotherapy** (*n* (%))			
Yes	31 (77.5)	28 (80.0)	3 (60.0)
No	9 (22.5)	7 (20.0)	2 (40.0)
**Previous experience with video conferencing** (*n* (%))			
Yes	19 (47.5)	17 (48.6)	2 (40.0)
No	21 (52.5)	18 (51.4)	3 (60.0)
**Musical background** (*n* (%))			
Music listening only	14 (35.0)	11 (31.4)	3 (60.0)
Playing one instrument	5 (12.5)	5 (14.3)	0 (0.0)
Playing several instruments	9 (22.5)	7 (20.0)	2 (40.0)
Singing in a choir	6 (15)	6 (17.1)	0 (0.0)
Dancing	6 (15)	6 (17.1)	0 (0.0)

Values are reported descriptively. Due to the small number of non-completers (*n* = 5), no inferential statistical comparisons were conducted

### Effectiveness

On the 5-point Likert scale on perceived helpfulness, participants reported a mean score of 4.77 (SD = 0.49). Of the 35 patients who completed the intervention, 28 (80%) rated it as “extremely helpful,” six (17.1%) as “very helpful,” and one (2.9%) as “somewhat helpful”. An analysis of participants’ open-ended feedback on the most beneficial components of the intervention revealed that shared music listening and discussion, reflection on personal musical biographies, the emotional impact of music, and the group experience—especially the sense of connectedness—were most frequently valued. Detailed qualitative results are provided in Online Resource 5.

Based on 35 complete data sets at T0 and T1, paired-samples t-tests showed significant reductions in PHQ-9 and GAD-7 scores immediately following music therapy (see Table 2), with medium to large effect sizes. In contrast, MDASI severity and interference scores showed no significant changes, with a slight descriptive increase in symptom burden.

**Table 2:** Results of paired-samples t-tests for standardized self-report measures at T0 and T1 (*n* = 35)

	T0		T1		*t*	*p*	Cohen’s ***dz*** [95% CI]
	Mean	SD	Mean	SD			
**PHQ-9**	12.00	3.92	9.37	4.08	4.02	< 0.001	0.68 [0.31, 1.05]
**GAD-7**	9.83	3.46	7.11	4.23	6.24	< 0.001	1.05 [0.64, 1.47]
**MDASI** severity	50.67	20.17	53.85	19.34	−1.18	0.502	−0.23 [−0.53, 0.14]
**MDASI** interference	29.93	13.27	31.44	11.93	−0.71	0.502	−0.14 [−0.45, 0.21]

*p*-values are adjusted using the Bonferroni-Holm method; effect sizes are reported as Cohen’s *dz* (0.20 = small, 0.50 = medium, and 0.80 = large effect)

*PHQ-9* Patient Health Questionnaire for Depression, *GAD-7* Generalized Anxiety Disorder Scale, *MDASI* M.D. Anderson Symptom Inventory

### Adoption

Although not formally assessed, contextual observations during the implementation process indicated implicit adoption by the institution, reflected in the provision of technical support and integration of the intervention into routine psycho-oncology services. Interest from other oncology centers further suggests potential for broader adoption.

### Implementation

A total of 35 out of 40 patients (87.5%) completed the intervention; five dropped out due to physical deterioration. Descriptively, non-completers tended to have a shorter duration of illness, were more frequently widowed, and reported no prior experience with group therapy compared to completers (see Table 1). Given the small number of non-completers (*n* = 5), these observations should be interpreted with caution. Mean session attendance was 90.1%, corresponding to an average of 7.2 out of 8 participants per session. Thirty-one patients (77.5%) attended at least seven sessions. Missed sessions were mainly due to medical appointments (70.8%), poor physical condition (16.7%), or private scheduling conflicts (12.5%).

Qualitative analysis of participants’ open-ended responses on suggestions for improvement and experiences with the online format revealed several key themes. Suggestions included extending the program, enhancing interaction during sessions, and strengthening connection between sessions. Reported challenges involved technical issues and limited personal contact, while perceived advantages included improved accessibility—especially for those with physical limitations or long travel distances—and easier integration into daily life and treatment routines (see Online Resource 5 for detailed overview of the qualitative results).

Analysis of therapists’ session protocols identified three main domains: perception of the online format, content and structure of the intervention, and its impact on patients’ daily lives (see Table 3). Therapists described initial technical challenges but increasing confidence over time. Despite the virtual setting, they observed strong group cohesion and emotionally supportive relationships. Shared musical experiences and personal musical reflections were seen as central to building connection and helping patients use music as a resource for emotional regulation and long-term coping.

**Table 3:** Therapists’ experience with the intervention—results of qualitative content analysis

Category	Sub-category	Code label	Example quote^a^
**Perception of the online format**	Technical aspects	Uncertainty due to technical problems (3)	*“I was very worried about whether the session would run smoothly after two participants’ microphones didn’t work.” (T1, S2)*
		Increasing confidence in technical handling (4)	*“Technical issues are becoming less frequent; all participants joined the virtual room without problems.“ (S, S3)*
	Group cohesion	Sense of closeness despite physical distance (18)	*“Participants reported thinking about other group members between sessions.” (T1, S4)*
		Experiencing appreciation and solidarity (13)	*“There was great appreciation for the group and its members, along with a sense of being understood.” (PS, S5)*
		Emotional openness within the group (6)	*“It was possible to express emotions (especially sadness and anger) and discuss them within the group” (T2, S6)*
	Therapeutic alliance	Challenges in perceiving emotional states (12)	*“It was more difficult to recognize emotional states: You can’t see very well on the screen if someone is crying.” (T1, S4)*
		Emotional depth of interaction (5)	*“I was impressed by how deeply participants engaged emotionally.” (T2, S5)*
**Content and structure of the intervention**	Listening to music together	Music triggers emotions (6)	*“Even in the first session, some participants showed unexpectedly strong reactions to the music they heard.” (T1, S1)*
		Music provides strength and energy	*“Many participants experienced the music as powerful and energizing.” (PS, S3)*
		Music evokes memories (4)	*“Participants reported that the music brought back touching memories (a feeling of comfort).” (T2, S4)*
	Presenting one’s own music	Music expresses personal narratives (6)	*“I was impressed by how the music chosen by the participants revealed very intimate aspects of themselves.” (T1, S4)*
		Sharing music builds connection (5)	*“Participants reported that presenting their music to others and talking about it created a strong sense of connection.” (T2, S7)*
	Biographical aspects	Music evokes positive life memories (7)	*“The musical biography evokes memories of happy phases of life associated with music in the participants.” (T2, S6)*
		Realizing the importance of music in life (6)	*“Participants reported that they realize how important music has been in their lives so far.” (PS, S7)*
		Sharing musical biographies in the group (6)	*“I felt that sharing musical biographies was actually the highlight for most participants.” (T1, S6)*
**Impact of the intervention on the patient’s everyday life**	Using music as a resource	Anchoring music in everyday life (11)	*“Participants thought about ways to integrate music into their daily lives.” (T2, S8)*
		Using music for emotional regulation (7)	*“Some participants reported that they created playlists for different moods (e.g., motivating, comforting, calming).” (PS, S6)*
	Altered approach to music	More intense and emotional experience of music (5)	*“One participant shared that she cried while listening to music for the first time in therapy, ‘but they were beautiful tears’.” (T1, S8)*
		Regaining emotional access to music (4)	*“I was touched by the feedback from some participants that they had distanced themselves from music for a long time but could finally listen again.” (T1, S7)*

*T1* therapist 1, *T2* therapist 2, *PS* psychology student, *S* session

^a^Translated from German; code label: the number in parentheses indicates the number of mentions

#### Maintenance

At T2, 27 participants completed the follow-up questionnaire. A loss-to-follow-up analysis comparing those with and without follow-up data revealed no systematic differences in demographic or clinical baseline characteristics, or in questionnaire scores at T0 and T1 (see Online Resource 6). The item on perceived effectiveness yielded a mean rating of 4.67 (SD = 0.47) on a 5-point Likert scale. Most participants rated the intervention as “extremely helpful” (74.1%), followed by “very helpful” (18.5%) and “somewhat helpful” (7.4%). The distribution mirrored T1 results, indicating stable perceptions of effectiveness over time.

Qualitative analysis of open-ended responses on long-term effects and integration into daily life indicated several lasting impacts. Participants reported increased acceptance of their illness and a more conscious way of handling emotions, often supported by the intentional use of music in everyday life. The intervention also appeared to foster a sense of belonging, with some participants remaining in contact after the intervention.

Repeated-measures ANOVAs showed significant reductions in PHQ-9 and GAD-7 scores from T0 to T1, which remained stable at T2. No significant time effects were found for MDASI outcomes. Detailed results are presented in Table 4.

**Table 4:** Results of repeated-measures ANOVA for standardized self-report measures (*n* = 27)

Outcome measure	T0 mean (SD)	T1 mean (SD)	T2 mean (SD)	*F (df*_*1*_*, df*_*2*_*)*	*p*	*η*^*2*^*ₚ*	T0-T1 (*p*)	T1-T2 (*p*)	T0-T2 (*p*)
**PHQ-9**	11.56 (3.96)	8.70 (4.03)	9.04 (4.19)	7.56 (2, 52)	0.009	0.37	2.85 (0.005)	−0.33 (0.28)	2.52 (0.02)
**GAD-7**	10.11 (3.61)	6.85 (4.56)	7.04 (4.32)	20.2 (2, 52)	< 0.001	0.62	3.26 (< 0.001)	−0.19 (0.16)	3.07 (< 0.001)
**MDASI **severity	50.63 (17.68)	54.16 (18.25)	52.68 (17.92)	0.70 (2, 52)	0.50	0.037	−3.53 (0.99)	1.47 (0.99)	−2.05 (0.12)
**MDASI **interference	29.14 (13.65)	30.9 (11.83)	32.19 (12.14)	1.82 (2, 52)	0.38	0.16	−1.76 (0.99)	−1.29 (0.30)	−3.05 (0.81)

*p*-values are adjusted using the Bonferroni-Holm method; effect sizes are reported as *η*^*2*^*ₚ* η^2^ₚ (≥0.01 = small, ≥0.06 = medium, and ≥0.14 = large)

*PHQ-9* Patient Health Questionnaire for Depression, *GAD-7* Generalized Anxiety Disorder Scale, *MDASI* M.D. Anderson Symptom Inventory

## Discussion

This study evaluated the implementation of a video-based group music therapy intervention for patients undergoing tumour therapy. Guided by the RE-AIM framework, the evaluation combined objective indicators with insights from participants and therapists, using quantitative and qualitative data. High enrollment and efficient recruitment indicate good reach and acceptability. Participants reported subjective benefit, accompanied by reductions in depression and anxiety symptoms. Shared musical experiences and emotional connectedness were particularly valued. Implementation was feasible despite minor technical challenges. Sustained benefits at follow-up and participants’ use of music for emotional regulation in daily life indicate maintenance after the intervention period.

Reductions in PHQ-9 and GAD-7 scores after the intervention reflect decreased depressive and anxiety symptoms, which remained stable at the three-month follow-up. Together, these findings suggest a potential stabilising role of music therapy during treatment, consistent with previous evidence on the psychological benefits of in-person music therapy in oncology [1–3]. At a conceptual level, this finding aligns with integrative models of music therapy that frame it as a supportive intervention targeting psychological well-being and coping with illness- and treatment-related stress [4].

Physical symptom burden is a central concern during tumor therapy. While prior research has reported increasing physical symptoms during chemotherapy [34], no significant changes were observed in MDASI severity or interference scores in the present study. This pattern suggests that video-based music therapy primarily supports psychological coping rather than directly modifying somatic symptoms. However, the absence of deterioration in physical symptom burden, alongside sustained improvements in anxiety and depressive symptoms, may indicate that the intervention primarily supports psychological coping with ongoing physical limitations rather than reducing somatic symptoms themselves.

A distinctive feature of our study design is the integration of both participant and therapist perspectives through qualitative feedback. This revealed two aspects of our intervention that were particularly impactful for both participants and therapists: sharing personally meaningful music and exploring its biographical significance. The effectiveness of biographical interventions in music therapy has been compellingly demonstrated in palliative care [35]. Our findings suggest that music-therapeutic biographical approaches may also be meaningful earlier in the cancer trajectory, supporting continuity of life narrative, highlighting music as a resource across different life phases, and fostering interpersonal connection among group members.

Therapeutic alliance and group cohesion are widely recognized as critical factors in online psychotherapy and videoconference-based music therapy [36, 37]. Challenges such as signal delays, lack of physical presence, and limited emotional attunement have raised concerns about the quality of therapist support [38, 39]. Although strong alliances can form in videoconference therapy, studies suggest they may develop more slowly and be less robust than in-person counterparts [40, 41]. Consistent with these observations, our findings revealed challenges such as therapists’ limited ability to perceive emotional states and reduced nonverbal resonance. Nonetheless, feedback from both participants and therapists indicated that therapeutic alliance was perceived as developing early and experienced as stable and containing throughout the intervention. Evidence on group cohesion in virtual formats remains limited; however, initial findings indicate that therapists must take a more active role in facilitating interaction and compensating for the absence of nonverbal cues [42, 43]. In the present study, participants emphasized the value of the group experience, particularly feelings of connectedness and belonging, which therapists similarly observed through early expressions of trust and openness. These study-specific observations suggest that group cohesion emerged rapidly in this intervention, potentially facilitated by the shared musical experience. Consistent with prior research on video-based psychotherapy and music therapy [39, 41, 44], our study identified technological and contextual challenges inherent to the online format. Core issues included unstable internet connections and uncertainty among participants unfamiliar with digital tools. Ensuring a private, distraction-free space introduced further difficulties, encompassing both data security and practical constraints at home. A preparatory, individual video session, in which participants practiced using the platform and discussed the importance of a secure setting, proved helpful in facilitating smooth group interactions. Moreover, real-time technical support enhanced the intervention’s feasibility. A psychology student provided technical assistance, contacting participants during connection issues and guiding re-entry via phone or email. Although disruptions were rare, the dedicated support increased participant confidence and enabled therapists to focus on group dynamics and therapeutic engagement.

### Limitations and future directions

Several limitations should be considered, particularly when comparing our findings with the existing music therapy literature, which has predominantly focused on in-person interventions. Given these differences in delivery format, group processes, and patient heterogeneity, direct comparisons with prior efficacy-focused studies are inherently limited. The sample consisted primarily of educated, female participants, which limits the generalizability of findings to more diverse patient populations. Moreover, heterogeneity in disease-related characteristics, including tumor type and disease stage (curative vs. palliative) encompassed a wide range of needs, illness experiences, and expectations. This diversity likely contributed to contrasting therapeutic foci, such as reintegration into daily life versus coping with advanced illness, thereby introducing greater variability in intervention content and participant responses. In addition, psychosocial and cultural factors, such as relationship status or religious and spiritual orientations, may influence individual coping processes and responses to music therapy. While relationship status was reported descriptively, religious or spiritual factors were not assessed in order to limit participant burden in a physically and emotionally strained population. The exploratory design, moderate sample size, and absence of a control group restrict the interpretability of effects beyond descriptive trends and precludes causal inference. Although the therapeutic alliance emerged as a prominent theme in qualitative feedback, it was not assessed using standardized, validated instruments. Finally, qualitative data were limited to written reflections collected via open-ended questionnaire items. Future studies should consider incorporating in-depth interviews or focus groups to capture more nuanced perspectives and explore underlying mechanisms of change. Building on these limitations, future research should evaluate the efficacy of video-based group music therapy in larger, controlled trials and examine differential effects across patient subgroups. Greater attention to psychosocial and cultural factors may help clarify their potential role in shaping therapeutic processes and outcomes. Combining qualitative approaches with standardized assessments of therapeutic relationship factors, such as the therapeutic alliance, could further strengthen interpretive validity and enhance comparability across studies, while supporting the sustainable integration of digital music therapy formats into multidisciplinary oncology care.

## Conclusion

This study suggests that video-based group music therapy is a feasible and acceptable intervention for patients undergoing tumour treatment. By reducing participation barriers related to mobility, fatigue, and geographical distance, the online format may enhance reach and support continuity of psycho-oncological care during a particularly demanding phase of illness. Participant and therapist feedback indicated the development of group cohesion and a stable therapeutic alliance, suggesting favourable conditions for implementation and adoption in an online setting. Preliminary indications of symptom relief, together with participants’ continued use of music in daily life, suggest potential benefits and transfer beyond the intervention. Taken together, the findings indicate that video-based group music therapy may represent a promising complement to existing psycho-oncological care.

## Supplementary Information

Below is the link to the electronic supplementary material.

ESM 1 PDF (65.5 KB)

ESM 2 PDF (91.4 KB)

ESM 3 PDF (186 KB)

ESM 4 PDF (135 KB)

ESM 5 PDF (169 KB)

ESM 6 PDF (167 KB)

## Data Availability

All data supporting the findings of this study are available within the paper and its Supplementary Information.
