# Supplementary material for: Implementing video-based group music therapy during cancer treatment: insights from a mixed-methods study
Source: Support Care Cancer. 2026 Mar 25;34(4):367. doi: 10.1007/s00520-026-10601-5 (PMC13018073; doi:10.1007/s00520-026-10601-5)
Supplement: Supplementary file 1 — PDF (65.5 KB) [file 520_2026_10601_MOESM1_ESM.pdf]

Online Resource 1: Good Reporting of A Mixed Methods Study (GRAMMS) Checklist (O'Cathain et al., 2008), outlining quality criteria for mixed methods study proposals and reports, including design, components, integration, and inferences, with emphasis on transparency in health services research.

Guideline	Section: Page
Justification to use a mixed methods approach to the research question	Methods – study design: p.4
Articulation of the design in terms of purpose, priority, and sequence of methods	Methods – study design: p.4
Describe each method in terms of sampling, data collection and analysis	Methods –Sample size and statistical analyses: pp.6-7
Delineate where and how integration occurs and who has participated in it	Methods – study design: p.4
Describe any limitation of one method associated with the presence of another	Discussion: p.14
Describe insights gained from mixing or integrating methods	Discussion: pp.13-14

Reference: O'Cathain A, Murphy E, Nicholl J. The quality of mixed methods studies in health services research. J Health Serv Res Policy. 2008;13: 92-98

Article Information:

Article title: Implementing Video-Based Group Music Therapy During Cancer Treatment: Insights from a Mixed-Methods Study

Journal name: Supportive Care in Cancer

Authors: Miriam Grapp, Charlotte Flock, Hans-Christoph Friederich, Till Johannes Bugaj

Corresponding author: Miriam Grapp, Department of General Internal and Psychosomatic Medicine, University Hospital Heidelberg, Germany, E-mail: miriam.grapp@med.uni-heidelberg.de
