# Supplementary material for: Implementing video-based group music therapy during cancer treatment: insights from a mixed-methods study
Source: Support Care Cancer. 2026 Mar 25;34(4):367. doi: 10.1007/s00520-026-10601-5 (PMC13018073; doi:10.1007/s00520-026-10601-5)
Supplement: Supplementary file 3 — PDF (186 KB) [file 520_2026_10601_MOESM3_ESM.pdf]

Online Resource 3: Music pieces listened to during therapy sessions. This table provides an overview of all music pieces listened to during therapy sessions, illustrating the diversity of musical material and associated biographical references. The list is descriptive in nature and does not represent a standardized intervention protocol.

Music piece (original title)	Composer / Performer	Genre	Selected by	Session	Group
Le quattro stagioni: La primavera, RV 269 (I. Allegro)	Antonio Vivaldi	Classical (Baroque)	Therapist	1	1, 3
Valse d'Amélie	Yann Tiersen	Film music	Therapist	1	2, 4-5
Imagining	Brian Crain	Contemporary instrumental	Therapist	1	1-5
Spring 2 (from *Recomposed by Max Richter: Vivaldi – The Four Seasons*)	Max Richter	Contemporary classical	Therapist	2	1-5
Nimrod (from *Enigma Variations, Op. 36*)	Edward Elgar	Classical (Romantic)	Therapist	2	1-5
Alegría	Cirque du Soleil	World / Soundtrack	Therapist	3	1-5
Skyline	Ola Gjeilo	Contemporary classical / Choral	Therapist	4	2,5
Goldberg Variations, BWV 988: Aria	Johann Sebastian Bach	Classical (Baroque)	Therapist	4	1, 3-4
Prelude in B minor, BWV 855a	Johann Sebastian Bach	Classical (Baroque)	Therapist	6	1-5
Music	John Miles	Rock / Pop	Therapist	8	1-5
La Mer	Charles Trenet	Chanson	Patient	3	1
Heast as net	Conchita Wurst & Ina Regen	Pop	Patient	3	2
What a Wonderful World	Louis Armstrong	Jazz / Pop	Patient	3	3
Ich bin ich	Rosenstolz	Pop	Patient	3	3
Liebe ist meine Rebellion	Frida Gold	Pop	Patient	3	4
Goodbye My Lover	James Blunt	Pop	Patient	3	4
Minor Swing (from *Chocolat*)	Django Reinhardt	Jazz / Gypsy jazz	Patient	3	5
Seeds of Growth	Malte Marten	Ambient / Handpan	Patient	3	5

Music piece (original title)	Composer / Performer	Genre	Selected by	Session	Group
Aquarela	Toquinho	Brazilian popular music (MPB)	Patient	4	1
Lichtjahre – Strandlied	Dragseth Duo	Contemporary folk / instrumental	Patient	4	1
Heroes	David Bowie (performed by The Ukulele Orchestra of Great Britain)	Pop (cover)	Patient	4	2
Take Me Home, Country Roads	John Denver	Country / Folk	Patient	4	2
Try to Remember	Harry Belafonte	Musical / Folk	Patient	4	3
With or Without You (Live at the Sphere, 2023)	U2	Rock	Patient	4	4
Stronger	Kelly Clarkson	Pop	Patient	4	5
Boogie Wonderland	Earth, Wind & Fire	Funk / Disco	Patient	5	1
Ain't Got No, I Got Life	Nina Simone	Soul / Jazz	Patient	5	1
Traditional Persian song	Homayoun Shajarian	Persian classical / Traditional	Patient	5	2
Can't Stop the Feeling!	Justin Timberlake	Pop	Patient	5	3
Dein Begleiter	Laith Al-Deen	Pop	Patient	5	4
Kraft	Glashaus	Pop / Soul	Patient	5	5
River Flows in You	Yiruma	Contemporary instrumental	Patient	6	1
Ain't Nobody	Chaka Khan	Funk / R&B	Patient	6	2
I Promised Myself	Nick Kamen	Pop	Patient	6	3
Eye of the Tiger	Survivor	Rock	Patient	6	4
Ich geh mit mir	LORI	Pop	Patient	6	5
Nessun dorma (from *Turandot*)	Giacomo Puccini (performed by Luciano Pavarotti)	Opera	Patient	7	1,4
The Good, the Bad and the Ugly (theme)	Ennio Morricone (Ukulele Orchestra version)	Film music	Patient	7	2
So schön	ROLA	Pop	Patient	7	3

Music piece (original title)	Composer / Performer	Genre	Selected by	Session	Group
Western	Premiata Forneria Marconi	Rock	Patient	7	5
O que é, o que é?	Gonzaguinha	Brazilian popular music (MPB)	Patient	8	1
Je suis malade	Lara Fabian	Chanson / Pop	Patient	8	2
Wenn ein Mensch lebt	Puhdys	Rock	Patient	8	3
Here's to the Heroes	Die 10 Tenöre	Crossover / Classical pop	Patient	8	4
Hymn	Barclay James Harvest	Rock	Patient	8	5

Article Information:

Article title: Implementing Video-Based Group Music Therapy During Cancer Treatment: Insights from a Mixed-Methods Study

Journal name: Supportive Care in Cancer

Authors: Miriam Grapp, Charlotte Flock, Hans-Christoph Friederich, Till Johannes Bugaj

Corresponding author: Miriam Grapp, Department of General Internal and Psychosomatic Medicine, University Hospital Heidelberg, Germany, E-mail: miriam.grapp@med.uni-heidelberg.de
