# Supplementary material for: Implementing video-based group music therapy during cancer treatment: insights from a mixed-methods study
Source: Support Care Cancer. 2026 Mar 25;34(4):367. doi: 10.1007/s00520-026-10601-5 (PMC13018073; doi:10.1007/s00520-026-10601-5)
Supplement: Supplementary file 4 — PDF (135 KB) [file 520_2026_10601_MOESM4_ESM.pdf]

Online Resource 4: Post-intervention questionnaire assessing participants' satisfaction and acceptance of the intervention at T1, based on an author-generated instrument. Original items were in German and translated into English for publication.

Dear participants,

Thank you for taking a few moments to answer the following questions on the evaluation of the video-based group music therapy intervention.

1. How helpful was the online music therapy group for you?

Not at all helpful	Slightly helpful	Somewhat helpful	Very helpful	Extremel y helpful
○	○	○	○	○

2. What did you particularly like about the music therapy online group?

____

____

____

____

____

3. What feedback or suggestions for improvement do you have for the video-based group music therapy?

____

____

____

____

____

4. What advantages do you see in online participation?

5. What challenges or difficulties do you see in the online format of the intervention?

Thank you very much for your participation and for sharing your experiences with us.

Article Information:

Article title: Implementing Video-Based Group Music Therapy During Cancer Treatment: Insights from a Mixed-Methods Study

Journal name: Supportive Care in Cancer

Authors: Miriam Grapp, Charlotte Flock, Hans-Christoph Friederich, Till Johannes Bugaj

Corresponding author: Miriam Grapp, Department of General Internal and Psychosomatic Medicine, University Hospital Heidelberg, Germany, E-mail: miriam.grapp@med.uni-heidelberg.de
