# Supplementary material for: Implementing video-based group music therapy during cancer treatment: insights from a mixed-methods study
Source: Support Care Cancer. 2026 Mar 25;34(4):367. doi: 10.1007/s00520-026-10601-5 (PMC13018073; doi:10.1007/s00520-026-10601-5)
Supplement: Supplementary file 5 — PDF (169 KB) [file 520_2026_10601_MOESM5_ESM.pdf]

Online Resource 5: Patients' experience with the intervention – results of qualitative content analysis of open-ended responses from post-intervention and follow-up questionnaires, including categories, codes, subcategories, and illustrative quotations.

**Category**	**Sub-category**	**Code label**	**Example quote[a]**
Intervention elements and aspects of the intervention found most beneficial	Structure and elements of the intervention	Guided imagery (6)	*"The mindfulness exercises at the start of each session."*
		Mindful music listening (10)	*"The conscious perception of the music and the feelings/memories that it evokes."*
		Talking about the music listened to together (9)	*"Talking about the emotions, memories and reactions with the others had both a calming and an encouraging effect on me"*
		Musical biography (9)	*"I have rummaged through my musical past and have often traveled back in time."*
	Effects related to the musical experience	Emotional activation (12)	*"I really liked that the emotional dimension was strongly addressed, which is often ignored in cancer therapy - when the body is in fight mode and has to function."*
		Music as a resource (6)	*"The sensible use of music and realizing how music can help in stressful situations."*
	Group experiences	Intensive exchange (14)	*"The opportunity to talk about music, dealing with the illness and about life in general."*
		Sense of connectedness through music (16)	*"It was a wonderful experience to see how unifying music can be and how a sense of community, even closeness, is created through music between people who were previously strangers."*
		Empathetic group leadership (8)	*"The therapists were present and empathetic, so I never had the feeling that I was being rushed or left alone."*
Feedback and suggestions for improvement	Duration and length of the intervention	Increasing the number of sessions (8)	*"The music therapy opened up so much for me that the 8 sessions were definitely not enough."*
		Continuation of the intervention (6)	*"Nevertheless, it would be wonderful to offer a continuation of the music therapy group, perhaps after a year."*
	Intensity of personal contact between participants	More time for exchange in the small groups (8)	*"Perhaps it would be possible to implement an 'online chill-out room' directly after each individual session so that you can stay together with the others in the group if you like."*
		Enabling contact among participants between sessions (8)	*"It might be nice to enable contact between participants so that you can stay in touch between sessions if you want to."*
		Higher proportion of music (4)	*"I would have liked a little more music in some sessions."*

	The role and function of music	Guide for the use of music (4)	*“It would be helpful to have a musical guide for emotional situations, e.g. when it might be useful to listen to which type of music.”*
Perceived benefits of online participation	Participation only possible due to online format	Participation possible even with severe physical limitations (20)	*“I wouldn't have been able to take part in the face-to-face format due to the severe side effects of the therapy.”*
		Participation possible despite long distance (10)	*“This meant that people who, like me, don't live near Heidelberg could also take part.”*
	Integration into daily and treatment routines	Stress relief in everyday life (12)	*“Better organization in everyday life, as the sessions can be easily integrated into the daily routine and with so many medical appointments during the week, it was a real benefit.”*
		Avoidance of longer journeys (10)	*“If we had met in person, I would have had a 2-hour drive to each location and I wouldn't have been able to do that.”*
	Aspects of safety and familiarity	No risk of infection (8)	*„This meant you could take part in the illness without infecting others, and you didn't have to constantly worry about being infected yourself.”*
		Familiar home environment (8)	*“I feel comfortable at home, so I was able to distance myself a little if things got too emotional”*
Challenges and difficulties of the online Format	Technical aspects	Problems making the connection (6)	*“The internet went down once when I tried to log into the session - it was quite stressful because I was briefly worried that I wouldn't be able to take part.”*
		Technical problems during session (10)	*“Sometimes there were sound problems during the meetings, so I didn't know if I was being heard.”*
	Interpersonal aspects	Impact on the relationship (4)	*“Perhaps flexibility and spontaneity suffer somewhat with online formats."”*
		Lack of personal contact (6)	*“I sometimes missed the personal contact and direct communication.”*
Long-term effects and transfer into everyday life	Changes in coping with the disease	Changed attitude towards the disease (8)	*“The music therapy group has helped me to change my view of my illness, to put it into perspective or to cope with it and to face the future, whatever it may be, with greater stability and clarity.”*
		More awareness and acceptance of one's feelings (6)	*“Music therapy has taught me to open up more space for my feelings and to take them more seriously.”*
	Implementing music as resource	Getting to know new music (14)	*“I have developed an open ear and curiosity towards less familiar music genres.”*
		Using music consciously in everyday life (14)	*”I have learned that music can be a valuable companion in everyday life and in a wide variety of life situations."”*

Sense of belonging	Connectedness with each other (18)	*"We didn't talk much about cancer, but I knew that my fears and hopes were understood, even without words."*
	Sustained contact among participants following the end of the intervention (14)	*"It's great that we've stayed in touch and continue to meet even after the intervention ended, a sign of how meaningful the group experience was for all of us."*
	The experience of being able to open up to others (8)	*"I had already listened to music attentively before, but doing it together with the others and being able to show it touched me in a special way and somehow also changed me."*

[a] Translated from German; Code label : The number in parentheses indicates the number of mentions.
The unit of analysis was the individual sentences. As a result, more than one statement was included in the analysis for those participants who had answered a question in more detail.

Article Information:

Article title: Implementing Video-Based Group Music Therapy During Cancer Treatment: Insights from a Mixed-Methods Study

Journal name: Supportive Care in Cancer

Authors: Miriam Grapp, Charlotte Flock, Hans-Christoph Friederich, Till Johannes Bugaj

Corresponding author: Miriam Grapp, Department of General Internal and Psychosomatic Medicine, University Hospital Heidelberg, Germany, E-mail: miriam.grapp@med.uni-heidelberg.de
