# Supplementary material for: Implementing video-based group music therapy during cancer treatment: insights from a mixed-methods study
Source: Support Care Cancer. 2026 Mar 25;34(4):367. doi: 10.1007/s00520-026-10601-5 (PMC13018073; doi:10.1007/s00520-026-10601-5)
Supplement: Supplementary file 6 — PDF (167 KB) [file 520_2026_10601_MOESM6_ESM.pdf]

Online Resource 6: Loss to follow-up analysis presenting baseline data for the total sample, intervention completers, and completers with and without follow-up data, including group comparisons using *t*-tests for continuous variables and chi-squared or Fisher's exact tests for categorical variables.

	**Total sample (n=40)**	**Intervention completers (n=35)**	**Completers with follow-up (n=27)**	**Completers without follow-up (n=8)**	**Test statistic** (completers with follow-up vs. completers without follow-up)	***p*-value**
**Age**						
Mean (SD)	53.12 (11.7)	53.77 (12.36)	54.59 (12.49)	51.00 (12.31)	$T(33) = -.72$	> .99
**Gender** n (%)						
Male	6 (15.00%)	5 (14.29%)	4 (14.81%)	1 (12.5%)	Fisher's exact test	> .99
Female	34 (85.00%)	30 (85.71%)	23 (85.19%)	7 (87.5%)		
**Residence** n (%)						
City	16 (40.00%)	14 (40.00%)	12	2 (25.00%)	Fisher's exact test	> .99
Rural area	24 (60.00%)	21 (60.00%)	15	6 (75.00%)		
**Duration of illnes**						
Mean (SD)	11.72 (	12.57 (9.03)	10.96 (6.23)	18.00 (14.42)	$T(7.79)^a = 1.34$	> .99
**Treatment intent** (n (%))						
palliative	23 (57.50%)	20 (57.14%)	15 (55.56%)	5 (62.50%)	Fisher's exact test	> .99
curative	17 (42.50%)	15 (42.86%)	12 (44.44%)	3 (37.50%)		
**Previous experience in group therapy** (n (%))						
yes	12 (30.00%)	12 (34.29%)	10 (37.04%)	2 (25.00%)	Fisher's exact test	> .99
no	28 (70.00%)	23 (65.71%)	17 (62.96)	6 (75.00%)		
**Previous experience in psychotherapy** (n (%))						
yes	31 (77.50%)	28 (80.00%)	22 (81.46%)	6 (75.00%)	Fisher's exact test	> .99
no	9 (22.50%)	7 (20.00%)	5 (18.52%)	2 (25.00%)		
**Previous experience with video conferencing** (n (%))						
yes	19 (47.50%)	17 (48.57%)	12 (44.44%)	5 (62.50%)	Fisher's exact test	> .99
no	21 (52.50%)	18 (51.43%)	15 (55.56%)	3 (37.50%)		

	Total sample (n=40)	Intervention completers (n=35)	Completers with follow-up (n=27)	Completers without follow-up (n=8)	Test statistic (completers with follow-up vs. completers without follow-up)	*p*-values
**Self-Report Measures** (Mean (SD))						
PHQ-9 (T0)	12.12 (3.78)	12.00 (3.92)	11.56 (3.95)	13.50 (3.63)	$T(33) = 1.24$	> .99
PHQ-9 (T1))	---	9.37 (4.08)	8.70 (4.03)	11.63 (3.62)	$T(33) = 1.84$	> .99
GAD-7 (T0)	10.10 (3.74)	9.83 (3.46)	10.11 (3.61)	8.88 (2.90)	$T(33) = -.89$	> .99
GAD-7 (T1)	---	7.11 (4.29)	6.85 (4.56)	8.00 (3.34)	$T(33) = .66$	> .99
MDASI severity (T0)	52.28 (20.47)	50.67 (20.17)	50.63 (17.68)	50.75 (26.61)	$T(33) = .01$	> .99
MDASI severity (T1)	---	53.85 (19.35)	54.16 (18.25)	53.13 (23.09)	$T(33) = -.15$	> .99
MDASI interference (T0)	31.28 (13.38)	29.93 (13.27)	29.26 (13.72)	31.50 (12.91)	$T(33) = .39$	> .99
MDASI interference (T1)	---	31.44 (11.93)	30.16 (12.18)	34.50 (11.49)	$T(33) = .86$	> .99

Abbreviations: PHQ-9, Patient Health Questionnaire for Depression; GAD-7, Generalized Anxiety Disorder Scale; MDASI, M.D. Anderson Symptom Inventory.
p-values are adjusted using the Bonferroni-Holm method
[a] Due to unequal variances, the Welch test was used.

<u>Article Information:</u>

Article title: Implementing Video-Based Group Music Therapy During Cancer Treatment: Insights from a Mixed-Methods Study

Journal name: Supportive Care in Cancer

Authors: Miriam Grapp, Charlotte Flock, Hans-Christoph Friederich, Till Johannes Bugaj

Corresponding author: Miriam Grapp, Department of General Internal and Psychosomatic Medicine, University Hospital Heidelberg, Germany, E-mail: miriam.grapp@med.uni-heidelberg.de
